# PLP1-Targeting Antisense Oligonucleotides Improve FOXG1 Syndrome Mice

**DOI:** 10.3390/ijms251910846

**Published:** 2024-10-09

**Authors:** Daniel C. S. Tan, Seonghee Jung, Yuanyuan Deng, Nicolle Morey, Gabriella Chan, Andre Bongers, Yazi D. Ke, Lars M. Ittner, Fabien Delerue

**Affiliations:** 1 Dementia Research Centre, Department of Biomedical Sciences, Faculty of Medicine Health and Human Sciences, Macquarie University, Sydney, NSW 2109, Australia; tancsdaniel@gmail.com (D.C.S.T.); seonghee.jung@mq.edu.au (S.J.); yuanyuan.deng@mq.edu.au (Y.D.); nicollemorey@gmail.com (N.M.); gabriella.chan1@outlook.com (G.C.); yazi.ke@mq.edu.au (Y.D.K.);; 2 Biological Resources Imaging Laboratory, University of New South Wales, Sydney, NSW 2052, Australia; andre.bongers@unsw.edu.au (A.B.)

**Keywords:** FOXG1 syndrome, neurodevelopmental disorder, mouse model, proteolipid protein 1 (PLP1), gene therapy, antisense oligonucleotides (ASOs)

## Abstract

FOXG1 syndrome is a rare neurodevelopmental disorder of the telencephalon, for which there is no cure. Underlying heterozygous pathogenic variants in the *Forkhead Box G1* (*FOXG1*) gene with resulting impaired or loss of FOXG1 function lead to severe neurological impairments. Here, we report a patient with a de novo pathogenic single nucleotide deletion c.946del (p.Leu316Cysfs*10) of the *FOXG1* gene that causes a premature protein truncation. To study this variant in vivo, we generated and characterized *Foxg1* c946del mice that recapitulate hallmarks of the human disorder. Accordingly, heterozygous *Foxg1* c946del mice display neurological symptoms with aberrant neuronal networks and increased seizure susceptibility. Gene expression profiling identified increased oligodendrocyte- and myelination-related gene clusters. Specifically, we showed that expression of the c946del mutant and of other pathogenic *FOXG1* variants correlated with overexpression of *proteolipid protein 1* (*Plp1*), a gene linked to white matter disorders. Postnatal administration of *Plp1*-targeting antisense oligonucleotides (ASOs) in *Foxg1* c946del mice improved neurological deficits. Our data suggest Plp1 as a new target for therapeutic strategies mitigating disease phenotypes in FOXG1 syndrome patients.

## 1. Introduction

FOXG1 syndrome is a rare neuro-developmental disorder (≈3 in 100,000 births [1]) for which there is currently no treatment or cure. Patients harbour pathogenic variants in *FOXG1*, the encoding gene for Forkhead box protein G1 (FOXG1), a protein exclusively expressed in the brain and testis. FOXG1 (also known as Brain factor 1 [2,3]) is a transcription factor involved in early embryonic development of the brain by controlling the fate of neuronal progenitors [4] and interneuron development [5]. Here, FOXG1 acts as a key regulator essential to the complex and finely tuned corticogenic process [6,7], a role that has been extensively documented in humans and mice alike [8,9,10,11,12,13,14,15]. FOXG1 syndrome patients generally present with severe physical and cognitive disabilities from birth, including microcephaly with atrophic Corpus Callosum, apraxia, generalized epilepsy [16], movement disorders and intellectual disability [17,18]. *FOXG1* function has, furthermore, been associated with autism spectrum disorder [19,20], glioblastoma [21,22], medulloblastoma [23,24] and hepatoblastoma [25]. In 2008, *FOXG1* variants were identified in patients with neurodevelopmental delay resembling Rett syndrome, hence describing the syndrome as a “congenital variant of Rett syndrome” [26]. Although both syndromes share common features [27], with potential interaction of the respective causative genes (i.e., *MECP2* and *FOXG1* [28]), clear phenotypic distinctions between FOXG1 and Rett syndromes have been delineated [29]. Genetically, FOXG1 syndrome is a congenital form of haploinsufficiency [30], with both the expression of *FOXG1* and its inheritance remaining to be completely elucidated. *FOXG1* is suspected to be imprinted in both mice [31] and humans [32], and most *FOXG1* disease-associated variants are inherited de novo (yet few cases of parental mosaicism have been reported [33]). Interestingly, genotype-phenotype analysis of FOXG1 syndrome identified more severe phenotypes for mutations within those *FOXG1* sequences that encode the N-terminal domain of *FOXG1* [34]. FOXG1 transcription factor interacts with several other proteins including Reelin [9], Grg6 [35], Smad [22], FGF [36], Pax6 [37], TLE1 [38], TLE2 [39], LHX2 [40], PLU-1 [41], as well as RNAs such as miR9 [42,43] and miR200 [44]. These FOXG1-centred networks are essential for the development, integrity and physiology of neurons, since FOXG1 regulates several neuronal receptors such as Androgen Receptors (ARs) [45] and Glutamate receptors [19]. However, a comprehensive analysis of all putative FOXG1 interaction partners and their integration in gene ontology networks and functional pathways is yet to be presented.

One of the main impediments in the study of FOXG1 syndrome is the absence of clinically relevant animal models. Knock-in lines carrying a partial or total disruption of the *Foxg1* gene by insertion of large transgenes such as Cre, Tet and LacZ [10] and a conditional knockout line [12] assisted in the study of FOXG1 function in vivo, but provided limited insight into disease mechanisms. Unfortunately, the insertion of exogenous transgenes to disrupt a gene can create unexpected and undesired effects [46,47,48]. As such, widespread ectopic expression has been documented in these *FOXG1* knock-in mice [49] and, despite attempts to moderate these shortcomings [50], classical gene-targeting approaches using ESC injections lead to chimerism, which often presents with unpredicted confounding effects [51], potentially generating inconsistent results [52,53,54,55].

In the present study, we report a new single nucleotide variant (SNV) in a patient with FOXG1 syndrome. We recapitulated this c.946del (L316Cfs*10) SNV deletion in mice using CRISPR/Cas9 genome editing of the murine *Foxg1* locus on a C57BL/6J background. To our knowledge, we generated the first clinically relevant mouse model of FOXG1 syndrome (hereafter referred to as c946del) and provide a comprehensive analysis of this mouse line, which recapitulates the hallmarks of its human counterpart, revealing new insight into the pathogenesis of FOXG1 syndrome.

## 2. Results

### 2.1. The c.946del Mutation Is Associated with Severe Cerebral Atrophy, Developmental Delay, Mental Retardation and Frequent Seizures in a FOXG1 Syndrome Patient

**Figure 1:**
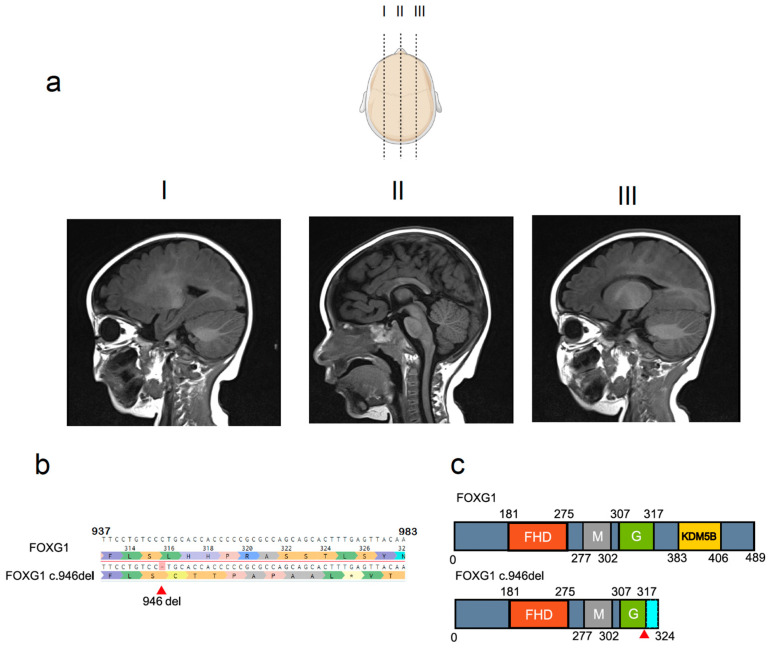
Genetics and clinical features of the c.946del mutation. (**a**) Brain T1-weighted magnetic resonance images of the c.946del patient’s brain reveal microcephaly, delayed myelination of the anterior portion of the corpus callosum and pachygyria. (**b**) Massively parallel sequencing (MPS) of the patient’s Foxg1 gene identified the c.946del mutation in Exon 1. (**c**) Structure of the human Foxg1 gene and its c.946del variant; forkhead box (FHD), mitochondria localization signal (M), Groucho binding domain (G) and KDM5B DNA methylase-binding domain (KDM5B) with the point mutation (red arrow) resulting in a frameshift (nine amino acids in cyan) leading to a C-terminus truncation which includes the KDM5B domain.

A male individuum (10 years old at the time of publication) was diagnosed with FOXG1 syndrome at 2 years of age following a screen for common neurodevelopmental disorder gene variants. The clinical symptoms were consistent with those of other FOXG1 syndrome patients [34]. The patient presented with infantile spasms, severe and refractory epilepsy, chorea and cortical visual impairment. Magnetic resonance imaging (MRI) revealed delayed myelination of the anterior portion of the corpus callosum, severe cerebral atrophy and pachygyria (Figure 1a). Massively Parallel Sequencing (MPS) for epileptic encephalopathy genes panel identified a de novo, heterozygous frameshift variant in the *FOXG1* gene. The c.946del (p.Leu316Cysfs*10) mutation (Figure 1b) was predicted to be pathogenic as it generates a truncated (324-amino-acid) FOXG1 protein, deleting a large part of its C-terminus, including the KDM5B domain (Figure 1c).

### 2.2. Generation of a Novel FOXG1 Syndrome Mouse Model

**Figure 2:**
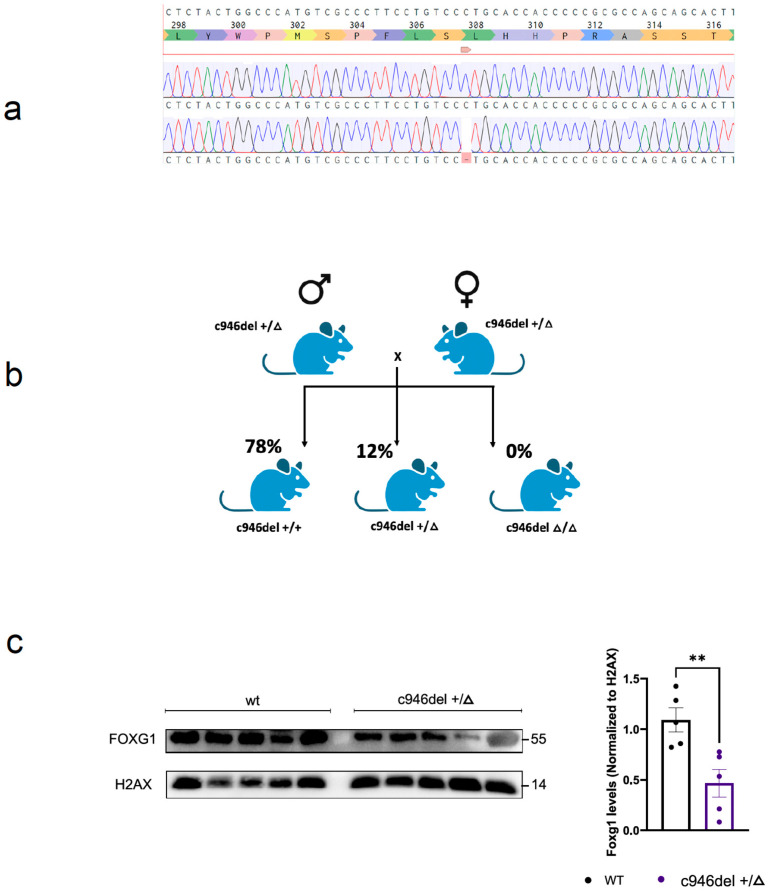
(**a**) Generation of c946del mice. Schematic of the genomic modification: a single cytosine deletion at position 922 (murine orthologue of the human c.946) in the Foxg1 gene was induced using CRISPR/Cas9 and confirmed by NGS and Sanger sequencing. (**b**) Breeding outcome of heterozygous c946del crossings: four breeding (five rounds) produced 18 × WT, 5 × c946del+/Δ (Het) and no c946del Δ/Δ (Hom) live pups. (**c**) Western blot analysis (brain extracts) of nuclear fractions shows a statistically significant reduction in protein expression in c946del mice compared with WT littermates. ** *p* < 0.01; Student’s *t*-test.

To investigate the functional consequences of the SNV, the c946del mutant mouse line was generated using CRISPR/Cas9 technology. C57BL/6J zygotes were electroporated [56] with a single guide RNA (sgRNA), High-Fidelity Cas9 protein to avoid off-targets [57], and a single stranded oligonucleotide (ssOligo) template carrying a single cytosine deletion at position 922 of the murine *Foxg1* (Supplementary Figure S1a,b), the homologue of the human c.946. Founders were identified by next-generation sequencing [58], and a male was selected to establish the colony. Transmission of the SNV was confirmed by Sanger sequencing (Figure 2a) and genotyping of the colony was performed by tetra primer-ARMS (T-ARMS)-PCR [59,60], producing a 551 bp mutant band and 394 bp wild-type (WT) band, respectively (Supplementary Figure S1c,d).

Backcrossing over more than five generations showed that heterozygous c946del+/Δ mice were viable and fertile, although the frequency of c946del+/Δ mice was below the expected Mendelian distribution of offspring from crossbreeding heterozygous mice (Figure 2b). This suggested a critical gene dosage of FOXG1 during embryonic development. Homozygosity of the SNV was embryonically lethal, as no homozygous c946delΔ/Δ mice were born. Therefore, viable heterozygous offspring were subsequently used for molecular and behavioural analysis at 1 and 3 months of age (Supplementary Figure S1e).

When determining FOXG1 protein levels in c946del mice, initial immunoblotting of brain extracts with a FOXG1 specific antibody showed no overt differences between c946del+/Δ and WT mice (Supplementary Figure S2a). However, enriching nuclear or cytosolic proteins from brains revealed a reduction of FOXG1 in nuclear fractions of c946del+/Δ mice compared with WT samples (Figure 2c). Taken together, introducing the human pathogenic c.946del variant into mice resulted in fully penetrant embryonic lethality of homozygous c946delΔ/Δ mice, while approximately half of the heterozygous c946del+/Δ mice were viable, with reduced nuclear FOXG1 levels.

### 2.3. Microcephaly and Functional Deficits in c946del Mice

**Figure 3:**
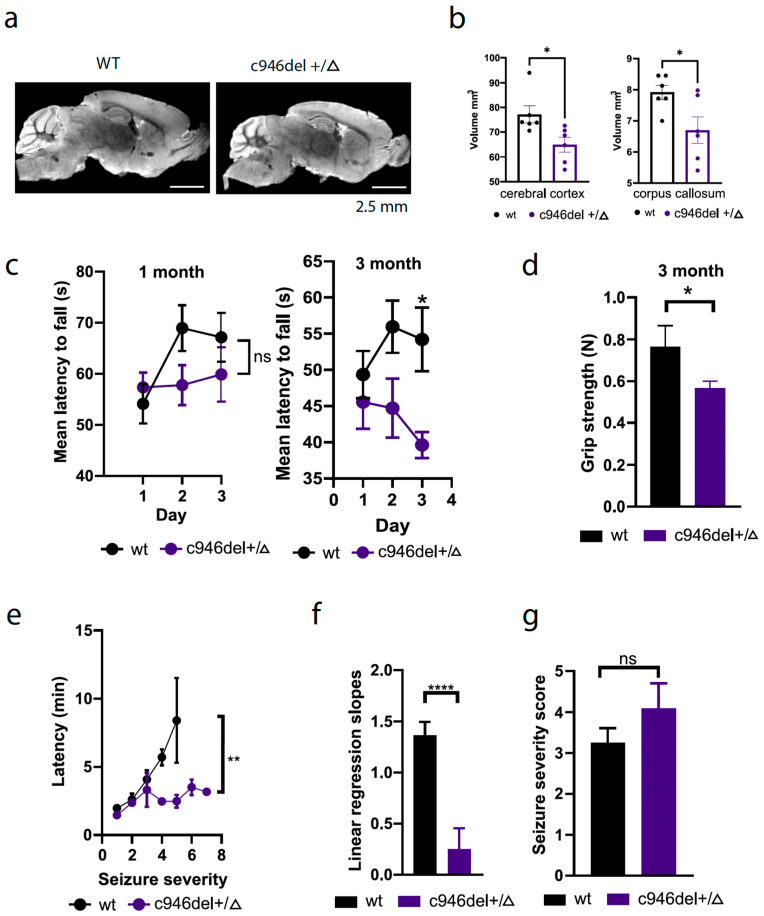
Microcephaly, functional deficits and increased seizure susceptibility in c946del mice. (**a**) Representative MRI images of wild-type and c946del heterozygous mouse brains. (**b**) Volumetric measurements of cerebral cortex and corpus callosum revealed a significant (~15%) reduction in c946del mouse brains. * *p* < 0.05, Student’s *t*-test; n = 6 WT, n = 6 c946del+/Δ; scale bar 2.5 mm. (**c**) The 3-month-old c946del+/Δ mice showed a significant reduction in mean latency, falling from the rotarod accompanied with a significant deficit in grip strength (**d**) in comparison to the WT controls. (**e**) c946del+/Δ mice showed faster progression to severe seizure stages in comparison to the WT controls. (**f**) Linear regression slopes showed significantly lower latency in reaching generalised seizure stages, although seizure severity (**g**) was comparable between WT and c946del+/Δ mice. * *p* < 0.05, ** *p* < 0.01, **** *p* < 0.0001, ns = not significant; Student’s *t*-test; Mann–Whitney test; 1-month-old mice (n = 10 WT, n = 9 c946del+/Δ), 3-month-old mice (n = 11 WT, n = 8 c946del+/Δ).

Reduced brain/cortex size (=microcephaly) with reduced and/or modified gyrification (pachygyria) are common features of FOXG1 syndrome, including in the above-reported c.946del patient (Figure 1a). Despite the lack of gyrification of the murine cortex, magnetic resonance imaging (MRI) of 3-month-old mouse brains revealed a global microcephaly in c946del+/Δ mice (Figure 3a). Accordingly, cortex and corpus callosum volumes were 15.7 ± 5.9% and 15.3 ± 6.1% smaller in c946del+/Δ mice than in WT controls, respectively (Figure 3b). Hence, the macroscopic brain structure of c946del+/Δ mice recapitulated features of the human FOXG1 syndrome.

Motor deficits are common hallmarks of FOXG1 syndrome [18]. Therefore, we assessed motor coordination and muscle strength of c946del+/Δ mice by subjecting them to rotarod and grip-strength testing, using standard protocols [61]. While there was only a non-significant trend toward shorter latency in falling off the accelerating rotarod in 1-month-old c946del+/Δ mice compared with WT controls, at 3 months of age c946del+/Δ mice showed significantly impaired motor coordination with decreasing fall latencies (Figure 3c). Furthermore, grip strength was significantly reduced in 3-month-old c946del+/Δ mice compared with WT littermates (Figure 3d). There were no differences in body weight or muscle atrophy in the c946del+/Δ mice compared to WT controls that would explain the observed differences. Accordingly, c946del+/Δ mice of both sexes were of comparable weights at 1 and 3 months of age (Supplementary Figure S2b). Similarly, laminin-stained cross sections of the soleus muscle showed no indication of muscular atrophy, with similar distribution profiles of fibre calibre in WT and c946del+/Δ mice (Supplementary Figure S2c). These data suggest impaired motor coordination and muscle function in c946del+/Δ mice.

Additional assessment of unrelated behaviours that have not been reported for FOXG1 syndrome individuals, such as changes in anxiety and activity as tested in the elevated plus maze and open field tests did not reveal overt abnormalities in c946del+/Δ mice compared to WT littermates (Supplementary Figures S3 and S4).

### 2.4. Spontaneous Epileptiform Neuronal Activity in c946del Mice

Seizures are frequent in FOXG1 syndrome [62]. To this end, we have not witnessed spontaneous overt convulsive seizure events in c946del+/Δ mice until 3 months of age. Therefore, we assessed their excitatory state by administering the chemo-convulsant pentylenetetrazol (PTZ) that induces excitotoxic seizures [63]. In line with our experience with this experimental approach [64], WT mice showed slow progression toward more severe seizure symptoms over time, but did not progress beyond the generalized clonus state (i.e., never displayed full body extension or reached *status epilepticus*) (Figure 3e–g). In contrast, 3-month-old c946del+/Δ mice progressed faster to severe seizure stages with significantly reduced latency, 63.6% of c946del+/Δ mice, but only 12.5% of the WT controls reaching generalised seizure stages. This suggested hyperexcitable neuronal networks in c946del+/Δ mice, resulting in increased seizure susceptibility.

**Figure 4:**
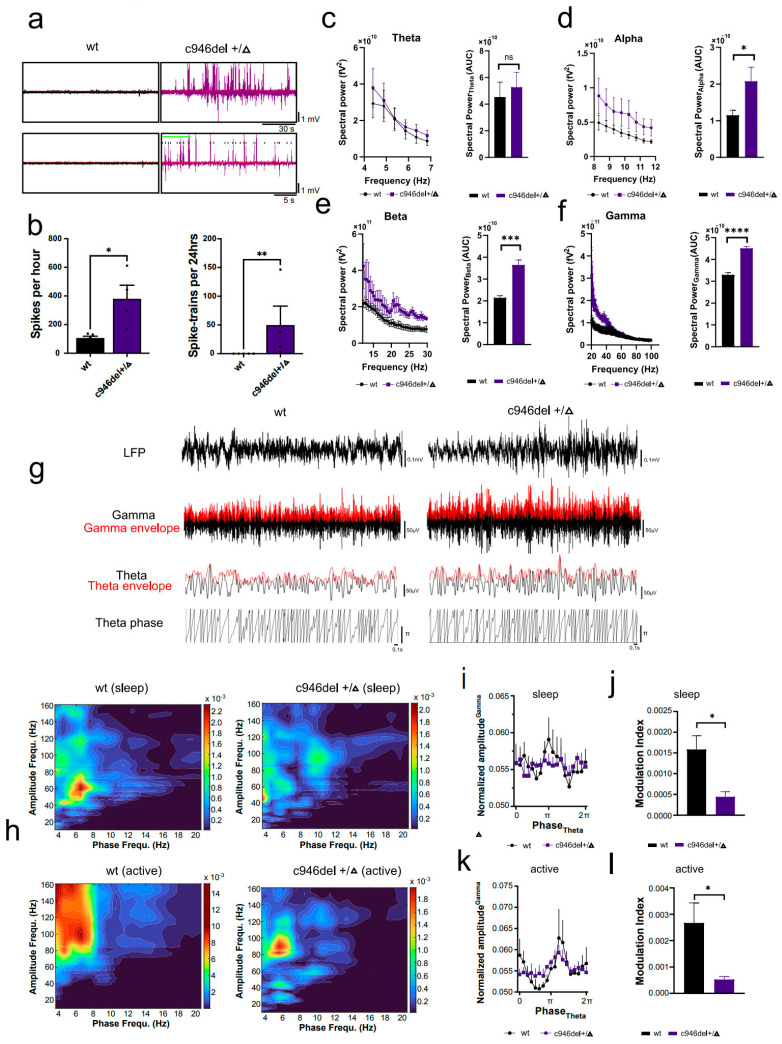
Spontaneous epileptiform neuronal activity in c946del mice. (**a**) Representative hippocampal electroencephalogram (EEG) recordings of 30 s segments from 3-month-old wild-type and c946del+/Δ mice. (**b**) Spike trains recorded over a 24 h period confirmed spontaneous epileptiform neuronal activity in c946del mice but not in WT controls. (**c**) Spectral power at theta frequencies (4–7 Hz) in WT and c946del+/Δ mice (left). Area Under the Curve (AUC) analysis of theta power (right). (**d**) Spectral power at alpha frequencies (8–12 Hz) in WT and c946del+/Δ mice (left) and corresponding AUC (right). (**e**) Spectral power at beta frequencies (12–30 Hz) in WT and c946del+/Δ mice (left) and corresponding AUC (right). (**f**) Spectral power at gamma frequencies (20–100 Hz) in WT and c946del+/Δ mice (left) and corresponding AUC (right). (**g**) Raw EEG (LFP = local field potential), bandpass filtered signals for gamma (20–100 Hz) and theta (4–12 Hz) oscillations, gamma amplitude and theta amplitude envelope (red) and theta phases in WT and c946del+/Δ mice. (**h**) Representative phase–amplitude comodulograms of hippocampal EEG recordings show aberrant coupling across multiple theta frequencies in both sleep and active stages in c946del+/Δ mice compared to WT controls. Lower theta phase–amplitude in c946del+/Δ mice compared to WT controls during sleep (**i**) and active (**k**) stages was accompanied with lower modulation indices (**j**,**l**). * *p* < 0.05, ** *p* < 0.01, *** *p* < 0.001, **** *p* < 0.0001, ns = not significant; Student’s *t*-test; n = 6 WT, n = 5 c946del+/Δ.

To assess neuronal network states directly in vivo, we implanted electrodes in the hippocampus of 3-month-old c946del+/Δ mice and WT littermates for telemetric electroencephalography (EEG) recording, in freely moving mice. Spontaneous discharges, spectral power density and cross-frequency coupling (CFC) for various wave frequencies were analysed using established protocols [65,66]. While WT mice presented with normal neuronal network activity during recordings, local Field Potentials (LFPs) of c946del+/Δ mice showed frequent spontaneous bursts of epileptiform spiking (Figure 4a). Accordingly, quantification of spontaneous epileptiform activity during 24 h EEG recording showed expected low numbers of isolated spikes and absence of spike strains in WT mice. In contrast, c946del+/Δ EEG recordings presented with significantly more spike trains per day (Figure 4b). This is in line with spontaneous and reoccurring epileptic brain activity in c946del+/Δ mice.

In the hippocampus, two main oscillations contribute to general synchronous network activity; pyramidal neurons generate theta oscillations [67] (4–12 Hz), while GABAergic interneurons contribute to gamma oscillations [68] (25–100 Hz). Both are interconnected when GABAergic interneurons synchronize pyramidal cells [69]. Reflective of this local network connectivity in the hippocampus, the phase of theta oscillations modulates the amplitude of gamma waves. This CFC of the theta-phase gamma amplitude is essential for cognitive performance, including in humans [70].

In c946del+/Δ mice, spectral power analysis of interictal EEG recordings revealed significantly greater power of alpha (8–12 Hz), beta (12–30 Hz) and low-frequency gamma (20–40 Hz) oscillations as compared to WT controls (Figure 4c–f). In addition, the phase and amplitude envelope of wake bandpass-filtered hippocampal recordings showed that c946del+/Δ mice had lower CFC between gamma amplitude and theta phase when compared to WT littermate recordings (Figure 4g). To further corroborate differences in phase–amplitude coupling, comodulograms were generated that scan frequency-band pairs of theta and gamma oscillations [71]. Analysis of CFC in both sleep and wake phases was performed to account for potential variances between these two consciousness states. Comodulograms of WT mice showed significant coupling around 2 Hz in the sleep stage and ~7 Hz in the wake stage. Conversely, CFC was disrupted, and aberrant coupling was observed across multiple theta frequencies in both sleep and active stages in c946del+/Δ mice (Figure 4h). Phase–amplitude distributions of the recordings obtained from WT and c946del+/Δ mice during both sleep and active phases showed significantly lower theta phase–amplitude in c946del+/Δ mice compared to WT controls during both sleep and wake (Figure 4i,k). Furthermore, the modulation indices (quantitative measures of frequency coupling [71]) were significantly lower in c946del+/Δ mice than in WT control recordings (Figure 4j,l), indicating uncoupled network oscillations in c946del+/Δ mice.

Taken together, hippocampal EEG recordings revealed spontaneous hypersynchronous epileptiform neuronal activity, together with aberrant network coupling, in c946del+/Δ mice that were hypersensitive to induced seizures.

### 2.5. Increased PLP1 Expression Levels and Oligogenesis in c946del Mice

**Figure 5:**
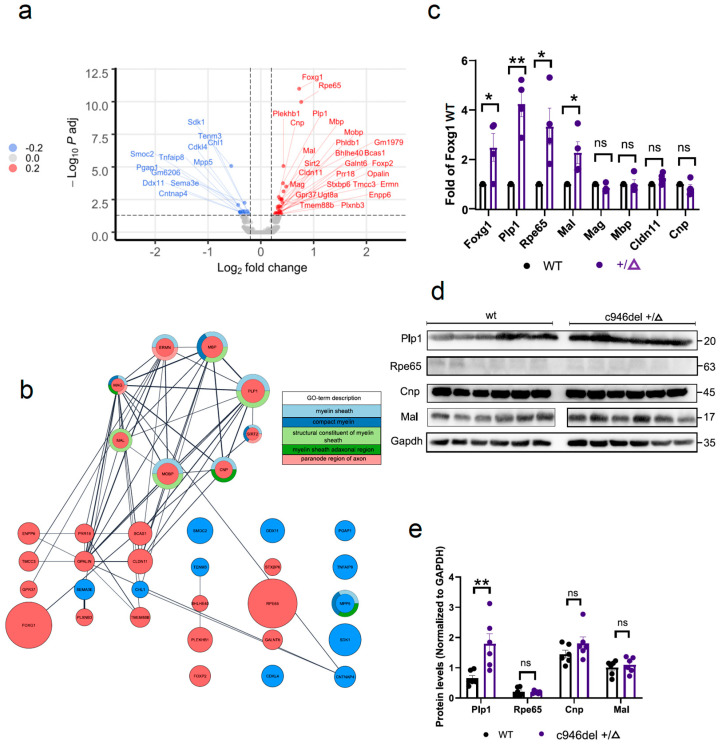
Increased PLP1 expression levels in c946del mice. (**a**) Volcano plot of differentially up-(red) and down-regulated (blue) mRNAs in the primary motor cortex of 3-month-old c946del+/Δ mice (n = 4) compared with WT (n = 4) littermates following RNA sequencing. (**b**) Protein–protein (STRING) analysis of differentially expressed in c946del+/Δ mice. Genes not linked to clusters are represented individually. Lines indicate protein–protein interactions; circle size correlates with expression level; red = upregulated; blue = downregulated; colour-coded rings relate to Gene Ontology annotations. (**c**) Quantitative PCR analysis of cortices for selected up-regulated genes in c946del+/Δ (n = 4) relative to WT controls (n = 4). (**d**) Western blotting for selected proteins in brain lysates revealed a significant increase (**e**) in PLP1 levels in c946del+/Δ (n = 6) mice compared to WT controls (n = 6). * *p* < 0.05, ** *p* < 0.01, ns, not = significant; Student’s *t*-test; one-way ANOVA followed by Tukey’s post hoc test.

To investigate whether the neuronal network deficits in c946del+/Δ mice were associated with changes in gene expression profiles, we performed RNA sequencing (RNA-seq) of the primary motor cortex from WT and c946del+/Δ mice. This revealed 39 differentially expressed genes (27 up- and 12 down-regulated genes with a false discovery rate [FDR] *p* ≤ 0.05) (Figure 5a and Supplementary Table S1). Unexpectedly, *Foxg1* was the most significantly upregulated gene. To gain functional insight from these candidate genes, the list of differentially expressed genes was subjected to STRING [72] (version 11.5) and identified a major cluster (8 of out 27 upregulated genes) associated with myelination and oligodendrocytes (Figure 5b). To validate these results in independent RNA extracts from cortices of WT and c946del+/Δ mice, we performed quantitative RT-PCR (qPCR). This confirmed significantly increased transcription of the four most up-regulated genes identified by RNA-seq (i.e., *Foxg1*, *Rpe65*, *Plp1* and *Mal*) in c946del+/Δ mice compared to WT controls (Figure 5c). At the same time, fewer up-regulated genes identified by RNA-seq (i.e., *Mag*, *Mbp*, *Cldn11* and *Cnp*) were not confirmed by qPCR. Similarly, eight downregulated genes as per RNA-seq (*Sdk1*, *Gap1*, *Smoc2*, *Tenm3*, *Cdkl4*, *Sema3e*, *Ddx11* and *Tnfaip8*) were not confirmed by qPCR (Supplementary Figure S5).

**Figure 6:**
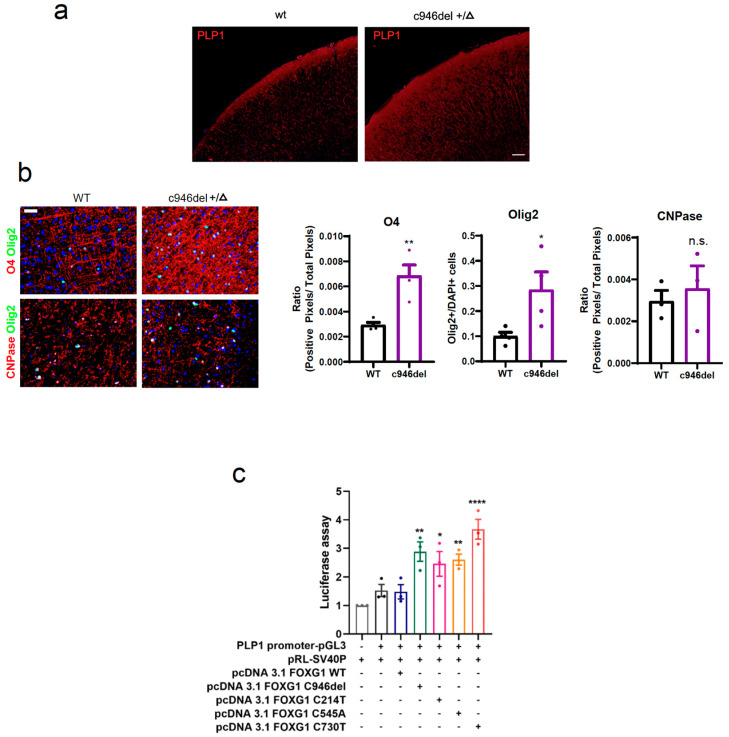
Increased oligogenesis in c946del mice. (**a**) Immunostaining on paraffin-embedded sections confirmed an increase in PLP1 expression in the cortex of c946del+/Δ mice compared to WT controls. Scale bar 100 μm. (**b**) Immunostaining of oligodendrocyte lineage markers O4, Olig2 and CNPase revealed an increase in early oligodendrocyte markers in the cortex of c946del+/Δ mice (n = 4) compared to WT controls. (n = 4). Scale bar 20 μm. * *p* < 0.05, ** *p* < 0.01, ns, not = significant; Student’s *t*-test. (**c**) PLP1 transcription reporter assay (Luciferase) revealed significantly increased PLP1 transcription by all FOXG1 variants (C214T, C545A, C730T and C946del) but not WT FOXG1. * *p* < 0.05, ** *p* < 0.01, **** *p* < 0.0001 (n = 3); one-way ANOVA followed by Tukey’s post hoc test.

Next, we determined the protein expression levels of the confirmed differentially up-regulated genes by Western blot analysis of 3-month-old mouse brain lysates for RPE65, PLP1 and MAL (and CNP) (Figure 5d). Of those, we only corroborated significantly increased levels of PLP1 protein in c946del+/Δ mice compared to WT controls, while RPE65, MAL and CNP were unchanged at the protein level (Figure 5e). In line with the Western blot findings, immunostaining of brain sections confirmed increased PLP1 expression in c946del+/Δ mice compared to WT controls (Figure 6a).

Since FOXG1 has been shown to inhibit gliogenesis [73], we next assessed whether the increase in PLP1 associated with the c946del variant of *Foxg1* is reflective of altered oligogenesis in the cortex of c946del+/Δ mice. Therefore, we stained brain sections of c946del+/Δ and WT mice with antibodies to oligodendrocyte lineage markers O4 (=oligodendrocyte progenitors), Olig2 (=oligodendrocyte differentiation) and CNPase (=mature oligodendrocytes). Interestingly, both O4 and Olig2 expression were significantly increased in the cortex of c946del+/Δ mice compared to WT control brains (Figure 6b), while CNPase labelling was comparable. Taken together, c946del+/Δ mice presented with increased *PLP1* expression at the RNA and protein level together with increased labelling of early oligodendrocyte markers.

### 2.6. Increased PLP1 Expression Is a Common Feature of FOXG1 Syndrome and a Potential New Therapeutic Target

The increased *PLP1* expression in c946del+/Δ mice raised the question as to whether this is a mutation-specific effect or a common result of pathogenic FOXG1 syndrome variants. Therefore, we designed a *PLP1* transcription reporter assay that consists of a vector encoding luciferase under control of the *PLP1* promoter, co-transfected with *FOXG1* variants in HEK293T cells. Beside the WT *FOXG1* and the *c.946del* variant, we tested three different previously reported mutations from the most comprehensive clinical report to date [34]; the nonsense mutant c.214 C > T (p.Q72*) located at the N-terminus, the missense mutant c.545 C > A (p.P182Q) located in the Forkhead conserved site and the missense mutant c.730 C > T (p.R244C) located in the Forkhead domain. While WT *FOXG1* had virtually no effect on *PLP1* transcription, all four pathogenic *FOXG1* variants markedly increased *PLP1* transcription (Figure 6c), suggesting a common molecular change in FOXG1 syndrome.

**Figure 7:**
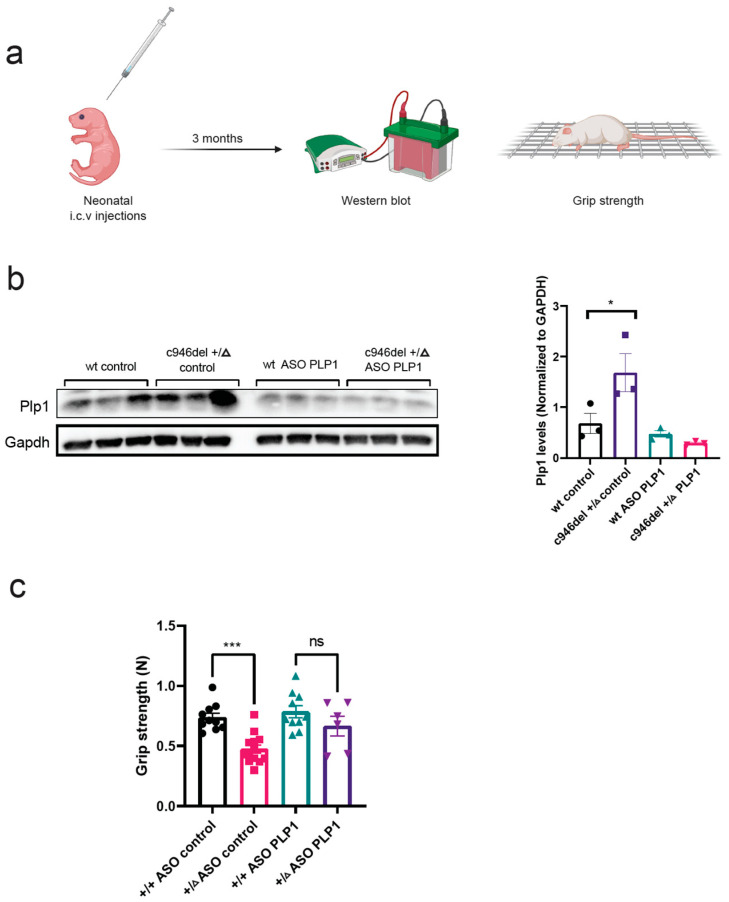
PLP1-targeting antisense oligonucleotide (ASO) therapy. (**a**) Workflow of the PLP1-targeting ASO therapy: intracerebroventricular (I.C.V) injection of newborn (P0) c946del+/Δ and WT mice followed by Western blot analysis and grip-strength test at 3 months of age. (**b**) Western blot analysis of whole brain extracts of c946del+/Δ mice and WT controls at 3 months of age. (**c**) Grip-strength test post ASO therapy on c946del+/Δ mice and WT controls at 3 months of age. * *p* < 0.05, *** *p* < 0.001, ns, not = significant; one-way ANOVA followed by Tukey’s post hoc test; n = 10 +/+ ASO control, n = 13 +/Δ ASO control, n = 10 +/+ ASO PLP1, n = 6 +/Δ ASO PLP1.

Duplication of the *PLP1* gene causes Pelizaeus–Merzbacher Disease (PMD), a rare X-linked CNS demyelination disorder [74]. Antisense oligonucleotide (ASO) therapy was previously shown to improve motor performance and extend the lifespan of PMD mice [75]. This prompted us to test *PLP1* as a potential therapeutic target for FOXG1 syndrome. A single intracerebroventricular (I.C.V) injection of *Plp1*-targeting ASOs was administered into newborn c946del+/Δ and WT mice (Figure 7a).

ASO treatment restored increased PLP1 expression in c946del+/Δ mice to levels of WT controls at 3 months of age (Figure 7b). Functional testing at this age revealed a grip strength of *Plp1*-ASO-treated c946del+/Δ mice that was no longer different from WT controls, contrasting with the significantly reduced grip strength of *control*-ASO-treated c946del+/Δ mice (Figure 7c). These results suggest that a single postnatal administration of *Plp1*-targeting ASO in c946del+/Δ mice can produce a sustained suppression of *Plp1* and consequently improve functional deficits.

## 3. Discussion

In the present study, we reported a novel de novo variant in *FOXG1*, c.946del, which resulted in a frameshift with a premature translational stop and caused FOXG1 syndrome in a male patient with severe CNS developmental and functional delay and frequent seizures. Based on this discovery, we generated a novel FOXG1-syndrome mouse line by introducing the homologue mutation in the murine *Foxg1* gene. In heterozygous c946del+/Δ mice, this resulted in reduced nuclear levels of the transcription factor FOXG1, microcephaly, functional deficits, spontaneous epileptiform discharges, and compromised oligodendrocyte function. Together, this recapitulated aspects of the human FOXG1 syndrome. For comparison, FOXG1 syndrome patients often present with developmental epilepsy [16,76], imbalance of excitatory/inhibitory neuronal expression [77], dyskinesia [17] or hyperkinetic movements and stereotypies [78]. Recently, phenotypic analysis of mice with heterozygous *Foxg1* deletion showed impaired neural plasticity, leading to social anxiety and cognitive defects [20,79]. Here, the c946del+/Δ line provides the opportunity for future comparison with heterozygous *Foxg1*-null mice to delineate the contribution of loss of FOXG1 function from toxic functions of *FOXG1* variant gene products in the pathogenesis of the syndrome.

Homozygous c946delΔ/Δ mice are embryonically lethal, in line with findings from homozygous *Foxg1* knockout mice [12]. While c946del+/Δ mice were viable and had no overt developmental deficits, we noted a lower than the Mendelian number of heterozygous c946del+/Δ offspring from crossing c946del+/Δ parents. This suggested a non-fully penetrant pre-birth lethal phenotype for the c946del+/Δ genotype, since we did not observe postnatal deaths. This highlights the importance of appropriate levels of FOXG1 protein at critical developmental stages, since timely and correct dosage of FOXG1 is essential for the normal development of the brain [80]. Absence of overt developmental deficits of surviving c946del+/Δ mice may indicate the presence of compensatory mechanisms. To what degree these are paralleled in humans remains to be established.

Microencephaly is accompanied with frequent epileptic events in the *FOXG1* c.946del patient, suggesting abnormal neuronal-network synchronicity. Similarly, c946del+/Δ mice present with frequent spontaneous epileptiform discharges during EEG recording, together with aberrant hypersynchronous neuronal-network activity during interictal recording periods. This indicates a defective interneuron network in c946del+/Δ mice, which is common in FOXG1 syndrome patients [19,20]. Disrupted and uncoordinated CFC in c946del+/Δ mice (including during sleep) suggests a dissociation of the neuronal network required for normal cognitive performance, in line with previously reported function of FOXG1 in mice [20,79]. To what degree the glial and white matter changes in c946del+/Δ brains contribute to the neuronal network aberrations remains to be shown.

Transcriptomics identified a major cluster of genes associated with myelination and oligodendrocytes in c946del+/Δ mice. At the protein level, we established an increase in PLP1 levels accompanied by a surge in early oligogenesis without terminal cell maturation in these mice. These results are consistent with recent findings showing that FOXG1 levels specifically decline during the neurogenic-to-gliogenic transition [81] and that FOXG1 is involved in myelination in vivo [82]. PLP1 is involved in synthesis, adhesion, compaction and wrapping of the myelin sheath [83]. As such, it is essential for the maintenance and survival of axons. It is also involved in the maturation of oligodendrocytes. Gene multiplication (predominantly duplications) that cause overexpression of the *PLP1* gene are linked to PMD, a rare X-linked CNS demyelination disorder [84]. Similarly, we describe overexpression of PLP1 as a new modality of FOXG1 syndrome. Early clinical reports of FOXG1 syndrome (prior to the discovery of the first *FOXG1* mutation) highlighted myelination defects [30], and such defects may be underdiagnosed [85], since white matter volumes are only assessed for the most severe cases [78]. Accordingly, delayed myelination due to increased PLP1 levels and subsequently compromised oligodendrocyte maturation may underly the delayed myelination of the corpus callosum in the c.946del FOXG1 syndrome patient reported in the present study. Importantly, experimental *Plp1*-targeting ASO therapy proved efficient in our c946del mouse model of FOXG1 syndrome. This suggests a potential novel therapeutic approach. To this end, treatments for leukodystrophies and white matter disorders might be repurposed for the treatment of rare neurodevelopmental disorders with myelination defects, such as FOXG1 syndrome.

## 4. Materials and Methods

### 4.1. Clinical Genetics

Massively parallel sequencing (MPS) was performed using the TruSight One panel (FC-141-1006) on an Illumina HiSeq 2500 (Ramaciotti Centre for Genomics, Sydney, Australia). Target regions of interest were restricted to coding regions and the canonical splice sites. Target rate indicated percentage of bases with a minimum depth of coverage of 20×. Alignments and variant calls were generated using SoftGenetics NextGene (State College, PA 16803, USA; version 2.3.4.2, accessed on 20 February 2015) with the February 2009 human genome assembly (GRCh37/hg19) and variant calls were limited to the requested panel of genes. Variants identified were accessed using Alamut Batch (version 1.2.0), and only variants with an allele frequency of <0.1% for dominant disorders, or <1% for recessive disorder, were reported. Sanger sequencing (AGRF, Westmead, Australia) was used to confirm pathogenic and likely pathogenic variants and/or to provide sequences for bases with insufficient coverage where 100% coverage is guaranteed for key genes. Variants of uncertain significance (VOUS) were reported but were not confirmed, and benign variants were not reported.

### 4.2. Generation of c946del+/Δ Mice

The generation of c946del+/Δ mice was carried out using our previously described electroporation method for the generation of genetically modified mice [86]. Briefly, a mixture of CRISPR reagents containing a High-Fidelity Cas9 protein (Alt-R^®^ *S.p.* Cas9, IDT), a sgRNA (Merck, Rahway, NJ, USA) targeting the first exon of the murine *Foxg1* gene (5′-CGCGGGGGTGGTGCAGGGAC-3′) and a single-stranded oligonucleotide (ssOligo) repair template were incubated at room temperature for 10 min to form RiboNucleoProtein (RNP) complexes. Fertilised C57BL/6J inbred fertilized zygotes were deposited in a 1 mm electrode (CUY501P1-1.5-Nepagene) in 3 μL of M2 medium. A total of 7 μL of RNP mix (final concentrations: 200 ng/μL Cas9, 450 ng/μL sgRNA and 600 ng/μL ssOligo) was added to the medium containing the zygotes. After electroporation, the zygotes were immediately washed in culture medium (KSOM) and reimplanted in pseudopregnant Swiss females. DNA extracted from tail biopsies of the pups underwent Sanger sequencing. Founders were identified by next-generation sequencing [58]. Out of five offspring, two carried the heterozygous c946del mutation, and a male was selected to establish the colony.

Mice of both genders were used throughout the study. Mice were housed in groups of 3–5 littermates in individually ventilated cages (Tecniplast, Varese, Italy) in a pathogen-free facility. The facility operated on a 12 h light/dark cycle (6 am–6 pm). Access to water and chow was ad libitum.

### 4.3. Genotyping

Genomic DNA isolated from tail biopsies of 3-week-old mice was extracted at 55 °C overnight in 300 μL of lysis reagent (50 mM Tris pH 8, 100 mM EDTA pH 8, 100 mM NaCl, 1% SDS, 0.5 mg/mL proteinase K) followed by standard isopropanol precipitation and purification [87].

Tetra-ARM PCR genotyping of Foxg1 c946del+/Δ was performed as previously described [59] using 0.1 μM of each inner primer (Inner forward: 5′-TCTACTGGCCCATGTCGCCCTTCCTGTACC-3′, Inner reverse: 5′-TGCTGCTGGCGCGGGGGTGGTGCAAGA-3′), 0.2 μM of each outer primer (Outer forward: 5′-TCGGGCCGGACGAGAAGGAGAAGGGC-3′, Outer reverse: 5′-CCGGGCGCTCATGGACGTGCTGGTCTG-3′), 200 μM dNTP, 1.25 mM of MgCl_2_, 5× Green Go*Taq* Buffer (Promega, Madison, WI, USA) and 1 U Go*Taq* polymerase (Promega). The reaction mix was placed in the thermocycler and incubated for 2 min at 95 °C, followed by 35 cycles of 1 min denaturation (95 °C), 1 min annealing (67 °C) and 1 min extension (72 °C), followed by an additional 5 min extension at 72 °C.

### 4.4. Magnetic Resonance Imaging (MRI)

MRI imaging of mouse brains was carried out as previously described [61]. Briefly, mice were anesthetized and transcardially perfused with phosphate-buffered saline followed by cold 4% paraformaldehyde (PFA). Brains and tissues were removed and post-fixed in 4% PFA overnight. Prior to imaging, the fixed brains were immersed in 0.2% *v*/*v* Gd-DTPA + 9 g/L NaCl/H_2_O solution for 24–48 h at 21 °C to reduce T1 relaxation time of the brain tissue. The brains were then transferred into a 1.3 mm ID, 2 mL Cryovial (Greiner, Frickenhausen, Germany) and submersed in Perfluoro-Polyether (Fomblin™ 6Y) for susceptibility matching. The Cryovial was mounted on the tip of a plastic tube, which was attached to the automatic positioning system of the MRI system. High-resolution anatomical imaging of the brain samples was performed in a 9.4-T Bruker BioSpec 94/20 Avance III micro-imaging system (Bruker, Ettlingen, Germany) which was equipped with BGA-12S HP gradients with maximum strength 660 mT/m and slew rate 4570 Tm/s and a dedicated 15 mm Quadrature Receive/Transmit RF-coil (Bruker, Ettlingen, Germany), to optimize the MR signal.

Images were acquired using an isotropic 3D Multi Gradient Echo (MGE) pulse sequence protocol optimized for small sample imaging. A total of 106 partitions were acquired in coronal slab orientation with 15 gradient echoes and echo-spacing of ∆TE = 3.45 ms. Otherwise, the protocol used the following major acquisition parameters: TR = 100 ms, First TE = 2.7 ms, ∆TE = 3.45 ms, #echos = 15, FA = 30°, FOV = 15 × 15 × 8 mm, matrix = 200 × 200× 106, Image Resolution = 75 μm^3^ (isotropic), Eff. Spectral BW = 78,125 Hz, total acquisition time with 2 ADC averages: 1 h and 10 min per specimen.

Imaging data were analysed using the medical imaging software package 3D Slicer (Version 4.10) [88]. Relevant anatomical structures, corpus callosum and cerebral cortex were segmented on the first echo image, and segmentation volumes were determined using functionality in the software [89].

### 4.5. Immunohistochemistry

All staining was carried out using established protocols [90]. Briefly, mouse tissues were first embedded in a gradient of Ethanol/Xylene/Wax cycles (Shandon Excelsior, Thermo Electron Corporation, Waltham, MA, USA) and then positioned onto cassettes (CASLID-01, Techno-plas). Then, 3–10 μm paraffin sections were obtained using a microtome (Shandon Finesse E, Thermo Electron Corporation) and subsequently mounted onto glass slides. The slides were dried at room temperature overnight and dehydrated for 2 h at 60 °C. Next, slides were submerged in xylene for 20 min at room temperature and rehydrated in decreasing concentrations of ethanol (100 → 70%). Slides were placed in tap water for 5 min, submerged into 10 mM citrate buffer, then heated in an antigen retrieval microwave (Milestone Medical) at maximum heat for 10 min. Slides were incubated with primary antibodies overnight at 4 °C, washed with 1xPBS before incubation with Alexa Fluor^®^ 488 conjugated anti-mouse antibody (A28175, Thermofisher, Waltham, MA, USA, 1: 250) and/or Alexa Fluor^®^ 555 conjugated anti-rabbit antibody (A27039, Thermofisher, 1: 250) in the dark for 1 h. Slides were then washed with 1xPBS and distilled water before being mounted with Fluoromount (SouthernBiotech, Birmingham, AL, USA) and imaged on an Axio Scan.Z1 slide scanner (Zeiss, Oberkochen, Germany). Primary antibodies used were those to mouse O4 (MAB345 Sigma Aldrich, Saint Louis, MO, USA; 1: 100) and rabbit Olig2 (AB9610 Sigma-Aldrich; 1: 100): Mouse CNPase (MAB1580 Sigma-Aldrich; 1: 100), Laminin (L9393 Sigma-Aldrich; 1: 100), and PLP1 (ab28486 Abcam; 1: 100).

### 4.6. Western Blotting

Immunoblotting and quantification of blots were performed as previously described [91]. Primary antibodies were those to FOXG1 [(N-terminal, Invitrogen, PA5-41493), MAL (Invitrogen, Waltham, MA, USA, MA5-32924), CNPase (Abcam, Cambridge, UK, ab6319), RPE65 (Abcam, ab13826), PLP1 (Abcam, ab28486), HNRNP (Sigma, R4653), Laminin (Sigma, L9393) and GAPDH (Millipore, Burlington, MA, USA, MAB374). Bound primary antibodies were detected with species-specific HRP-labelled secondary antibodies and visualized with HRP substrate (Millipore) on a Chemidoc imager (Bio-Rad, Hercules, CA, USA). Quantification was carried out using ImageJ (NIH, Bethesda, MD, USA).

### 4.7. Nuclear–Cytosolic Extraction

Frozen tissues were homogenised in 10 μL/mg of nuclear–cytosol buffer (10 mM HEPES, 10 mM NaCl, 1 mM KH_2_PO_4_, 5 mM NaHCO_3_, 5 mM EDTA, 1 mM CaCl_2_, 0.5 mM MgCl_2_) with a Dounce homogeniser. Samples were incubated at 4 °C for 10 min in rotation. Subsequently, 0.5 μL/mg of 2.5 M sucrose buffer was added and mixed into the homogenate. The mixture was centrifuged at 6300× *g* for 10 min at 4 °C and the supernatant was collected as the cytosolic fraction. The pellet was resuspended in 1 mL TSE buffer (10 mM Tris (pH 7.2), 300 mM sucrose, 1 mM EDTA, 0.1% IGEPAL) and centrifuged at 4000× *g* for 5 min at 4 °C. This step was repeated 3 times, discarding the supernatant each time. The pellet was then resuspended in 2 μL/mg RIPA buffer before rotating at 4 °C for 30 min. Finally, the resuspension was centrifuged at 14,000 rpm for 10 min at 4 °C and the supernatant was collected as the nuclear fraction.

### 4.8. Quantitative Real-Time PCR

Transfect HEK293T cells (American Type Culture Collection—ATCC, Manassas, VA, USA) were homogenized in 1 mL TRIzol reagent (Thermofisher). RNA was isolated from the aqueous phase of the TRIzol:chloroform mixture and purified with the RNeasy Mini Kit (Qiagen). The RNA mixture was purified by DNase 1 (Qiagen, Venlo, The Netherlands). The cDNA synthesis kit used was the SuperScript^®^ VILO™ kit (Invitrogen). Quantitative real-time PCR (qPCR) was next performed on the ViiA 7 qPCR machine (Applied Biosystems, Waltham, MA, USA) with Power SYBR Green PCR master mix (Applied Biosystems) and primers for each target gene (Supplementary Table S2). The mean of two replicates (for each sample) was used to establish the threshold cycle (Ct). The Ct was the cycle number at which the fluorescence signal was significantly greater than the background fluorescence. The difference between the Ct value for the target gene and GAPDH (ΔCt = Ct (target gene) − Ct (GAPDH)) was calculated for each sample. The efficiency (E) of the reaction was determined by the slope of the standards (E = 10^−1^/slope). The relative level of gene expression for each sample was E − ΔCt.

### 4.9. Luciferase Assay

Site-directed mutagenesis was performed on a full-length (FL) FOXG1 expression vector (human cDNA) to generate the clinically relevant c.214, c.545, c.730 and c.946 mutations using the QuickChange Site-Directed Mutagenesis Kit (Agilent, Santa Clara, CA, USA), following the manufacturer’s recommendations. Primers used for each mutation are included in Supplementary Table S3.

Transcriptional activity of FOXG1 was measured using the Promega dual-luciferase reporter assay system (E1960; Promega, Madison, WI, USA), according to the manufacturer’s instructions. Briefly, vectors were transfected into HEK293T cells (American Type Culture Collection—ATCC), using polyethylenimine (PEI). A total of 2.5 μg of DNA and 15 μL of PEI were added to saline, to a final volume of 500 μL. DNA was allowed to incubate for 20 min before being added dropwise to each cell-culture plate and harvested 48 h later. Control vectors were p5X-pGL3empty and p5X-pPLP1. Expression vectors were pcDNA3.1-FOXG1(FL), pcDNA3.1-FOXG1(C214T), pcDNA3.1-FOXG1(C545A), pcDNA3.1-FOXG1(C730T) and pcDNA3.1-FOXG1(C946del), respectively. Luciferase assay was performed on a PHERAstar microplate reader (BMG Labtech, Ortenberg, Germany). The *Renilla* luciferase expression was normalized to the *Firefly* luciferase expression.

### 4.10. Rotarod

All behavioural tests have been carried out as per Ke et al. [61]. Motor coordination of mice was assessed on a five-wheel Rotarod treadmill (Ugo Basile) in accelerating mode (5 to 60 rpm in 2 min). Each mouse was subjected to five trials over three days. For each day, the average time the mouse stayed on the wheel was recorded.

### 4.11. Grip Strength

Mouse grip strength was assessed with a grip strength meter (Chatillon, AMETEK, Berwyn, PA, USA). Mice were positioned on a thin metal wire attached to the meter, with a double overhand grip. To measure grip strength, mice were pulled away from the meter horizontally, until grip was lost. The peak force (N) was recorded. The average force was recorded after five attempts.

### 4.12. Elevated Plus Maze

Anxiety and disinhibition behaviours were assessed on the elevated plus maze (Ugo Basile, Lombardia, Italy). The maze consisted of two opened and two closed arms (each 35 cm by 5.5 cm) and a centre platform (5.5 cm by 5.5 cm) elevated 60 cm above the ground. Mice were first placed in the room for 1 h before testing for acclimatisation, then placed on the edge of the open arm. All movements were video captured for 5 min and analysed using the AnyMaze tracking software (Stoelting Co., Wood Dale, IL 60191, USA).

### 4.13. Open Field

Locomotor activity was assessed in an open field chamber. Briefly, mice movements were recorded for 10 min after placing each mouse at the corner of a 40 × 40 cm Perspex box in an enclosed cupboard. The box was divided into an outer and inner zone, with the inner zone consisting of a 17.5 × 17.5 cm square in the centre of the box. Videos of mice movements were analysed using the AnyMaze software version 7.45 (Stoelting Co., Wood Dale, IL 60191, USA).

### 4.14. Electroencephalogram (EEG) Recordings

Implantation of EEG electrodes on remote telemetric transmitters (DSI) was performed as previously described [92]. Briefly, an aseptic incision exposing the midline of the scalp of each mouse (under ketamine/xylazine anaesthesia) was performed before securing the head in a stereotactic frame (Kopf Instruments, Tujunga, CA, USA). The bregma was located and bur holes (0.05 mm) were created using a bone micro drill (Fine Science Tools, F.S.T., Foster City, CA, USA) above the hippocampus (ML +2.0, AP −2.0, DV −2.0 mm, relative to bregma). Recording electrodes connected to a single-channel telemetric transmitter (ETA-F10, DSI) were positioned in the hippocampus while the reference electrode was inserted above the cerebellum (ML 0.0, AP −6.0, DV 0.0 mm from bregma). The transmitter was secured in a subcutaneous pouch on the back of the animal. Polyacrylate was used to secure electrodes in position before wound closure. EEG recordings were collected with a DSI wireless receiver setup (DSI) with amplifier matrices using the Ponemah (v6.41.20418.1, DSI) recording software at 500 Hz sampling rate [92]. Movement artefacts were screened manually in all recordings and only artefact-free EEG passages were included in the analysis. Noise filtering of raw LFP was performed using a powerline noise filter (Neuroscore, DSI).

EEG recordings were next analysed using the NeuroScore software v3.0 (DSI) with an integrated spike and sleep–wake cycle detection module. Spike trains and sleep–wake activity were detected and automatically assigned. All spectral analyses of signal power at individual frequencies were performed with the integrated fast Fourier transform (FFT) spectral analysis function of Neuroscore. Frequency bands of theta, alpha, beta, and gamma waveforms were defined as 4–7 Hz, 8–12 Hz, 12–30 Hz and 20–100 Hz, respectively. Spectral contributions of theta, alpha, beta and gamma waves were quantified by area-under-curve (AUC) analysis across the defined frequency band in artefact- and hyper-synchronous spike-free sequences (1 min in length). Theta phase and gamma amplitude cross-frequency coupling (CFC) was analysed using MATLAB, as previously described [71].

### 4.15. PTZ-Induced Seizures

Excitotoxic seizures were induced by intraperitoneal injection of pentylenetetrazole (PTZ) at 50 mg/kg body weight, as previously described [93]. Briefly, 10 min after injection, seizure progression was scored using the following paradigm: 0, no seizures; 1, immobility; 2, tail extension; 3, forelimb clonus; 4, generalized clonus; 5, bouncing seizures; 6, full-body extension; and 7, *status epilepticus*, and the time to reach each stage was recorded.

### 4.16. RNA Sequencing

RNA sequencing (RNAseq) of primary-motor-cortical brain samples from 3-month-old c946del+/Δ mice (n = 4) and their WT littermates (n = 4) was performed. mRNA extraction and sequencing were carried out by Macrogen INC. (Seoul, Korea). Briefly, mRNA extraction was performed using the Maxwell^®^ 16 LEV SimplyRNA tissue kit (Promega). Library construction used TruSeq Stranded Total RNA with the Ribo-Zero Gold sample prep kit and TruSeq rapid SBS kit. Sequencing was performed on an Illumina HiSeq 2500 platform, following the HiSeq 2500 System User Guide (#15035786 v01 HCS 2.2.70 protocol) using HCS version 2.2 as sequencing control. For read mapping, HISAT2 [94] was used to align paired reads against the GRCm38 (mm10) mouse reference genome. The resulting bam files were visualised in the Interactive Genomics Viewer [95]. To identify differentially expressed genes (DEGs), Kallisto [96] pseudo-alignment against the GRCm38 (mm10) reference genome was used to generate transcript-per-million (TPM) values. TPM values for WT and c946del+/Δ mice were then compared using DESeq2 software [97], with an adjusted *p*-value threshold of 0.05 to identify DEGs. The volcano plot of DEGs was created using R Studio.

### 4.17. ASO Therapy

*Plp1*-targeting ASO therapy in postnatal mice was performed, as previously described [75]. Briefly, pups at P0 were administered 30 μg of either Plp1-targeting ASOs or control (non-targeting) ASO. ASOs were administered with a NanoFil microsyringe (World Precision Instruments) by intracerebroventricular (I.C.V.) injections of cryoanaesthetized mice. The needle was inserted to a depth of 2 mm and positioned at 0.25 mm lateral to the sagittal suture and 0.50–0.75 mm rostral to the neonatal coronary suture [98]. A total volume of 2 μL was injected into the left ventricle. Mice were placed on a heating pad to recover, and subsequently reintroduced to the dam.

### 4.18. Statistical Analysis

Statistical analysis was performed using GraphPad Prism 13 software (GraphPad). Statistical tests used for each analysis are indicated in the respective figure legends. All values are presented as mean ± standard error mean (SEM).

## Data Availability

The data that support the findings of this study are available from the corresponding author upon reasonable request. The c946del mouse line is freely accessible to all non-profit research organisations.

## Supplementary Materials

The following supporting information can be downloaded at: https://www.mdpi.com/article/10.3390/ijms251910846/s1.
