# Supplementary material for: PLP1-Targeting Antisense Oligonucleotides Improve FOXG1 Syndrome Mice
Source: Int J Mol Sci. 2024 Oct 9;25(19):10846. doi: 10.3390/ijms251910846 (PMC11477415; doi:10.3390/ijms251910846)
Supplement: Supplementary file 1 [file ijms-25-10846-s001.zip › ijms-3241222-supplementary figures.pdf]

Tan et al. Supplementary Figures

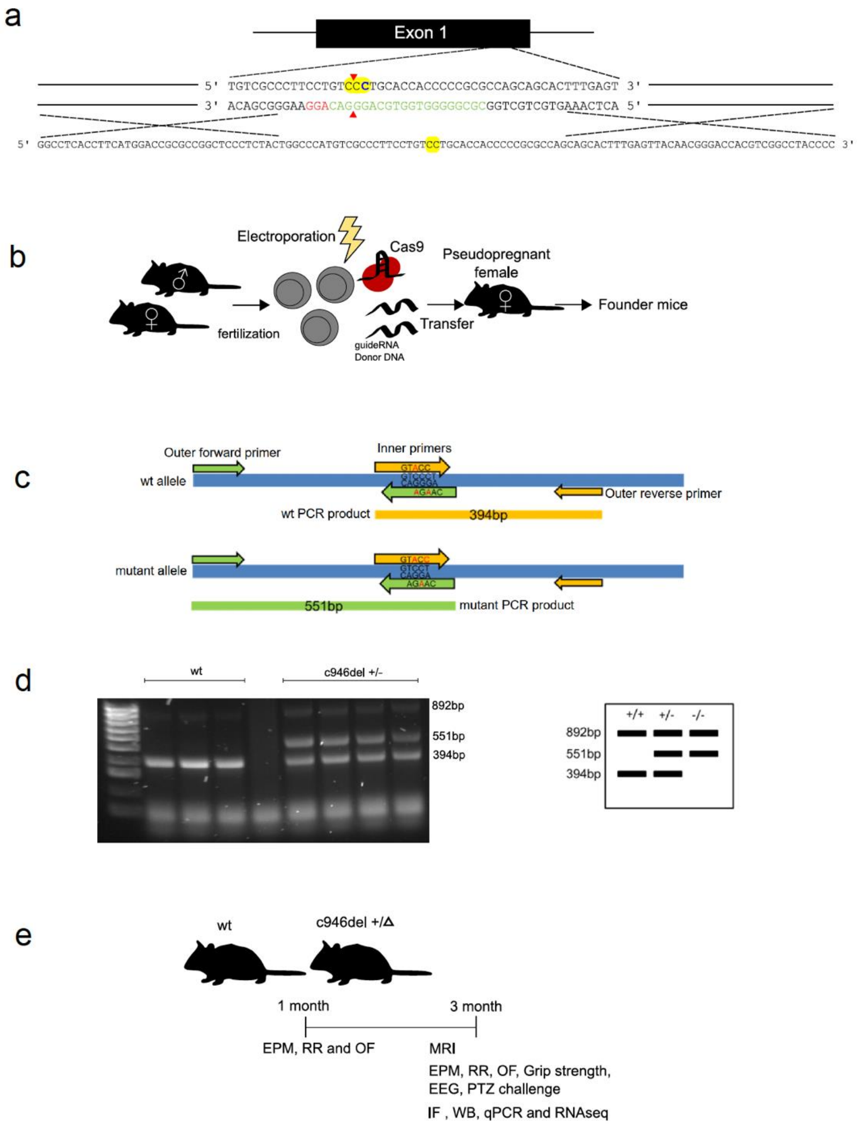

Supplementary Figure 1. Genome editing, genotyping and phenotyping strategies to characterize the c946del mice; (**a**) Schematic of the genome editing strategy: the Cas9-sgRNA RNPs (green, protospacer adjacent motif in red) induce double-stranded breaks induced by Cas9 (red arrows). Homologous Directed Repair (HDR) using a ssOligo (bottom) results in the deletion of a cytosine at position 922 (blue). (**b**) Workflow to generate the mutant mice: C57BL/6J zygotes were electroporated in medium containing the Cas9 RNP complexes and repair template before reimplantation into pseudo-pregnant females. (**c**) Schematic of the primers used for the Tetra-ARMS PCR and resulting WT (394bp) and mutant (551bp) bands. (**d**) Illustrative electrophoresis gel for WT and Heterozygous c946 mice. (**e**) Phenotypic characterization: mice underwent elevated plus maze (EPM), Rotarod (RR) and Open field (OF) tests at 1 and 3 months of age. Grip strength, electroencephalogram (EEG) and pentylenetetrazole (PTZ) challenges together with immunofluorescence (IF), western blot (WB), quantitative PCR (qPCR) and RNA sequencing (RNAseq) were carried out on 3-month-old mice.

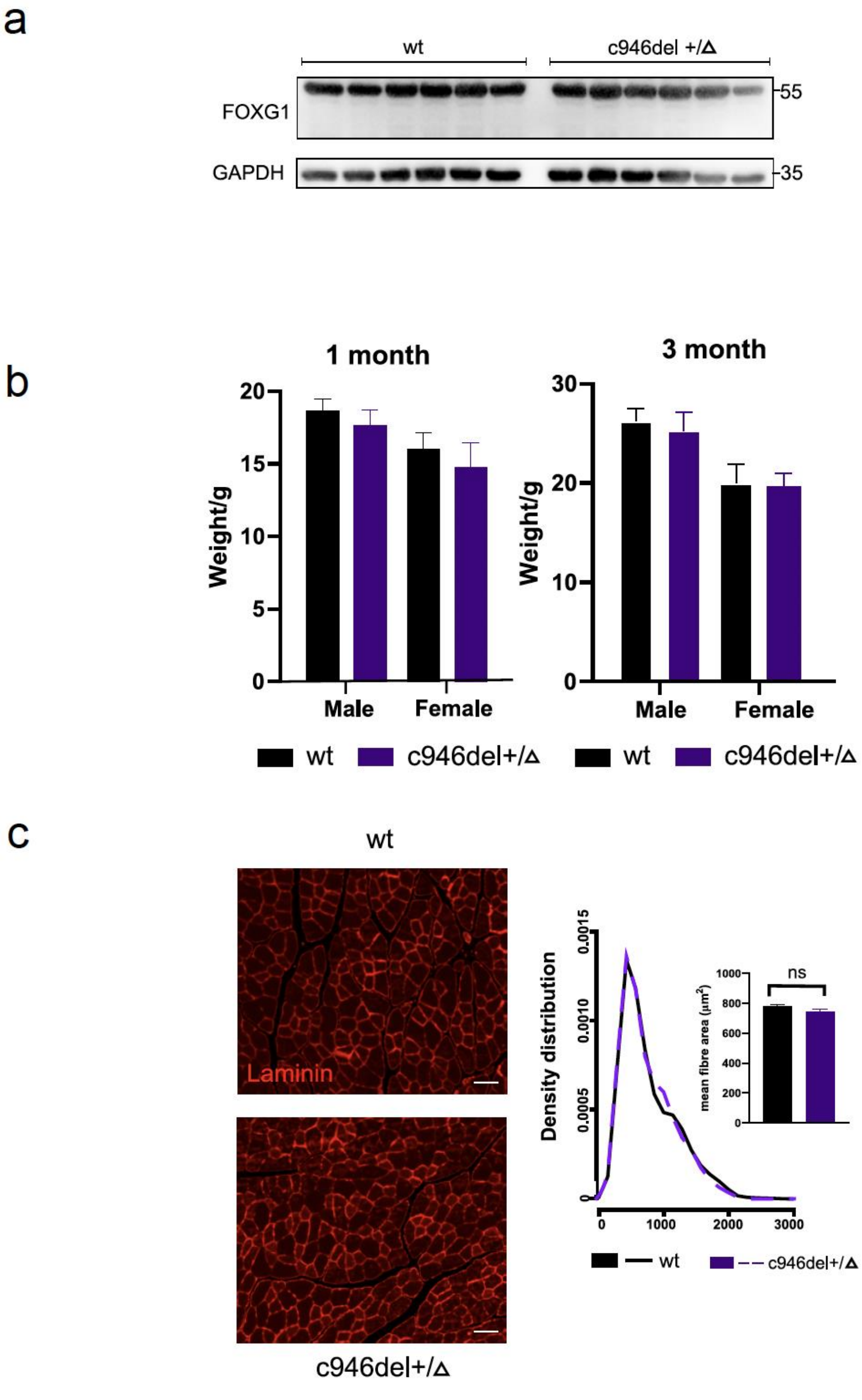

Supplementary Figure 2. Functional deficits in c946del mice are not correlated with muscle changes; (**a**) Immunoblotting of mouse WT controls and c946del+/D brain lysates using an N-terminal FOXG1 specific antibody. (**b**) Body weight of c946del+/D mice and WT littermates at 1 and 3 months of age. (**c**) Laminin-stained cross sections of the soleus muscle showed similar distribution and no muscular atrophy in c946del+/D mice. (*Scale bar 50μm).*

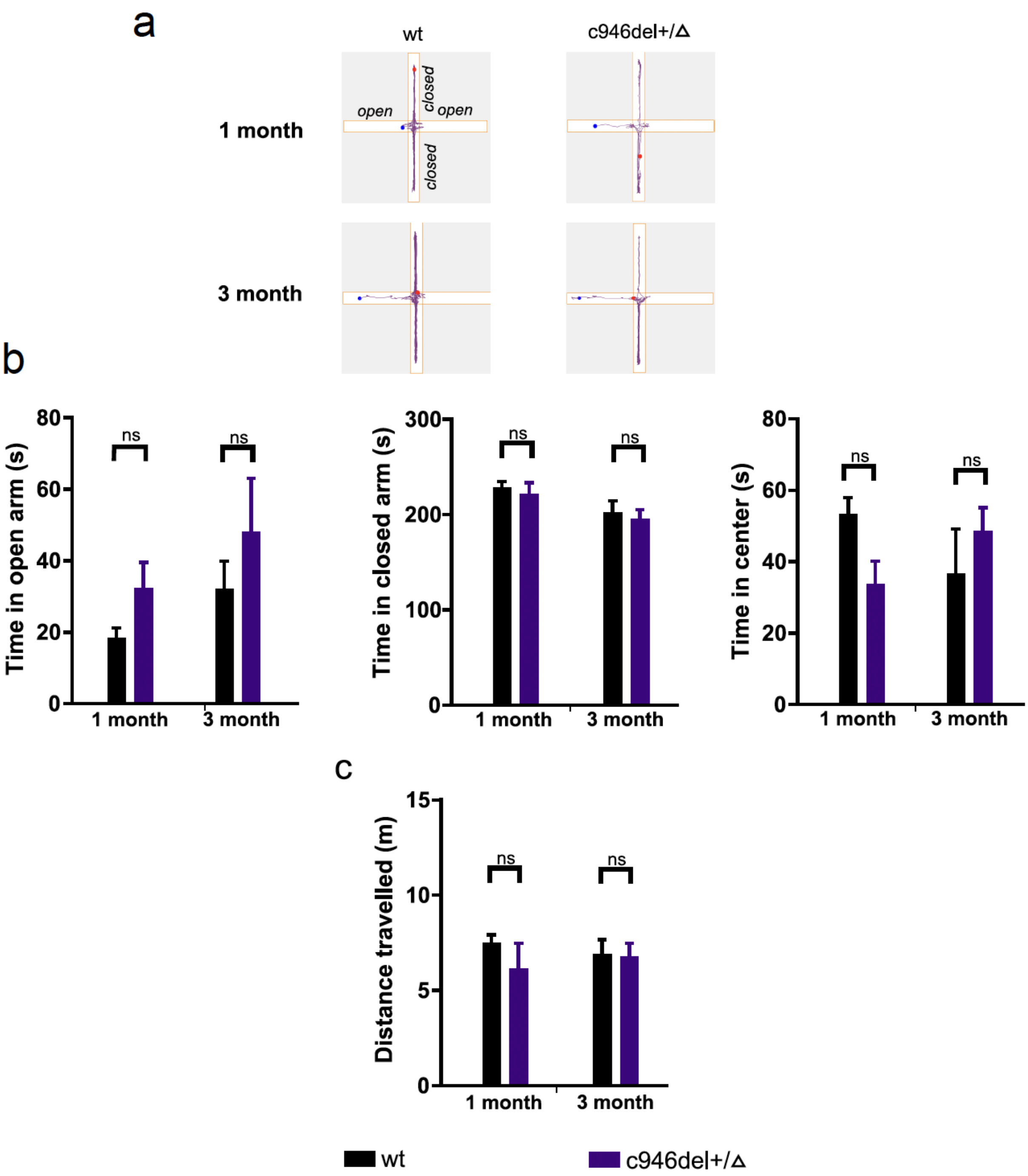

Supplementary Figure 3. No anxiety or disinhibition-like behaviour in c946del mice; (**a**) Representative traces of 1-month-old and 3-month-old WT and c946del+/D mice; in the elevated-plus-maze (EPM) which comprises two open and two closed arms. (**b**) Quantification of time spent in the open arms, closed arms, and centre. (**c**) Quantification of total distance travelled in the EPM. *ns*, not significant; student *t*-test; 1-month-old mice (n = 10 WT, n = 9 c946del+/D), 3-month-old mice (n = 11 WT, n = 8 c946del+/D).

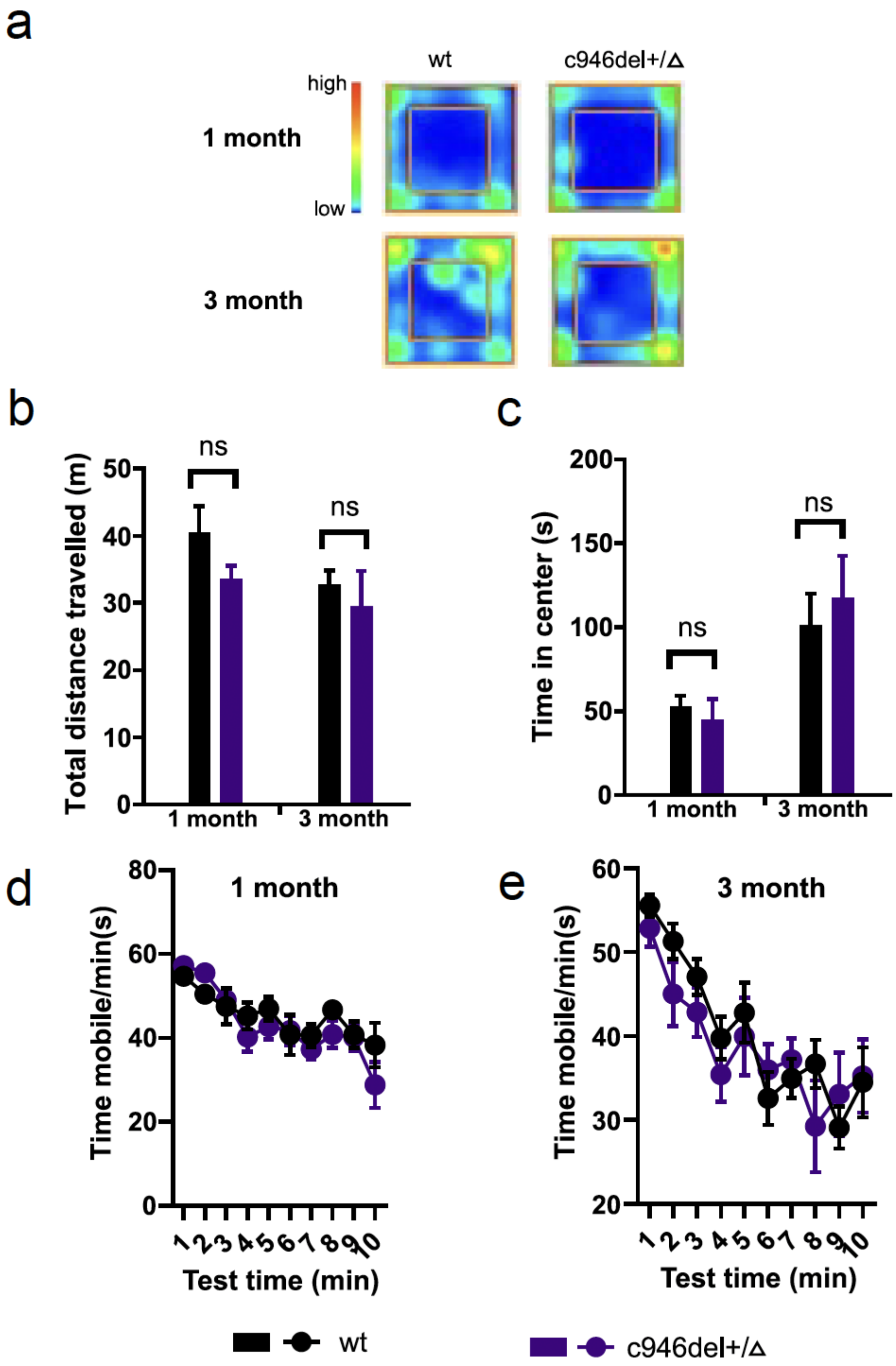

Supplementary Figure 4. No changes in locomotor activity in c946del mice; (**a**) Representative heat maps of 1-month-old and 3-month-old WT and c946del+/D mice in the open field. Trace intensity represents the amount of time spent in the region of the open field. (**b**) Quantification of total distance travelled over 10 min. (**c**) Quantification of time spent in the centre zone. (**d**,**e**) Locomotor activity for each minute over a 10min period; No significant differences were observed across all mouse groups. *ns*, not significant; student *t*-test; 1-month-old mice (n = 10 WT, n = 9 c946del+/D), 3-month-old mice (n = 11 WT, n = 9 c946del+/D).

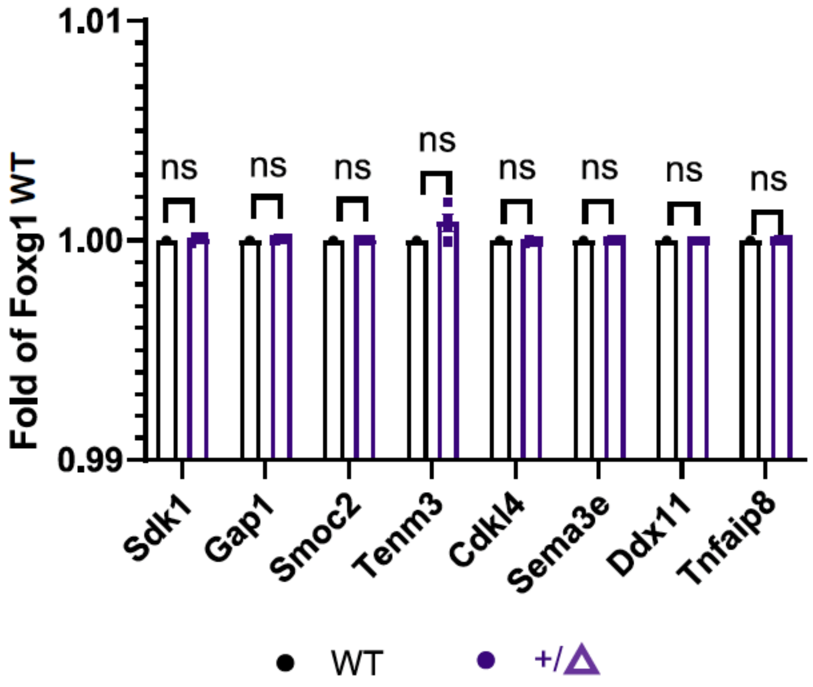

Supplementary Figure 5. qPCR analysis of selected downregulated genes in c946del mice; Quantitative PCR analysis of cortices for selected downregulated genes in c946del+/Δ (n = 4) relative to WT controls (n = 4).
