# Supplementary material for: PLP1-Targeting Antisense Oligonucleotides Improve FOXG1 Syndrome Mice
Source: Int J Mol Sci. 2024 Oct 9;25(19):10846. doi: 10.3390/ijms251910846 (PMC11477415; doi:10.3390/ijms251910846)
Supplement: Supplementary file 1 [file ijms-25-10846-s001.zip › ijms-3241222-supplementary tables.pdf]

Supplementary Table 1. List of differentially regulated cortical genes of c946del mice at 12 weeks of age. Gene expression profiling by RNA sequencing and DESeq2 analysis with a cut-off of false discovery rate [FDR] $p \leq 0.05$ revealed 39 differentially regulated genes (27 up- and 12 down-regulated genes). Values are given as log2 fold changes of WT controls.

GeneID	Base Mean	Log2FoldChange	StdError	Wald Stats	*p* Value[2]	*p* Value
Foxg1	941.6769	0.724986	0.089796	8.073664	6.82E-16	1.02E-11
Rpe65	94.5014	0.763695	0.099229	7.696316	1.40E-14	1.05E-10
Plekhb1	1853.219	0.427759	0.071553	5.978238	2.26E-09	8.47E-06
Sdk1	787.6882	−0.55963	0.093271	-6.00006	1.97E-09	8.47E-06
Plp1	14699.96	0.419315	0.077368	5.41973	5.97E-08	0.000179
Gm1979	70.9857	0.479637	0.090801	5.282314	1.28E-07	0.000319
Cldn11	1327.44	0.428517	0.084121	5.094052	3.50E-07	0.000752
Cnp	4269.452	0.340142	0.069733	4.877803	1.07E-06	0.002015
Mbp	20258.94	0.393058	0.0824	4.770109	1.84E-06	0.002866
Tmem88b	638.1411	0.362778	0.076166	4.762967	1.91E-06	0.002866
Mobp	9685.945	0.392584	0.08323	4.716861	2.40E-06	0.003271
Bcas1	1495.313	0.411647	0.089793	4.584398	4.55E-06	0.005577
Pgap1	1988.504	−0.30716	0.06718	−4.57221	4.83E-06	0.005577
Smoc2	410.1836	−0.42768	0.095534	−4.47674	7.58E-06	0.008134
Ermn	835.0247	0.346538	0.07846	4.416722	1.00E-05	0.010037
Mal	2995.974	0.318173	0.072881	4.365661	1.27E-05	0.011201
Foxp2	979.2471	0.375988	0.086096	4.36706	1.26E-05	0.011201
Gpr37	1132.782	0.323181	0.07555	4.277689	1.89E-05	0.015762
Tmcc3	789.2629	0.357506	0.086143	4.150121	3.32E-05	0.024585
Tenm3	4269.707	−0.25455	0.061659	−4.12843	3.65E-05	0.024585
Cdkl4	419.971	−0.34163	0.082889	−4.12153	3.76E-05	0.024585
Opalin	340.537	0.361953	0.087475	4.137807	3.51E-05	0.024585
Sema3e	909.1342	−0.30936	0.074325	−4.16227	3.15E-05	0.024585
Ddx11	184.9525	−0.39107	0.095997	−4.07376	4.63E-05	0.0278
Tnfaip8	202.8102	−0.39712	0.097483	−4.07378	4.63E-05	0.0278
Gm6206	55.51003	−0.38776	0.096352	−4.02444	5.71E-05	0.033002
Mag	2500.013	0.277918	0.06926	4.012702	6.00E-05	0.033402
Bhlhe40	1655.474	0.306261	0.076879	3.983694	6.79E-05	0.035152
Cntnap4	1019.418	−0.32913	0.082603	−3.9845	6.76E-05	0.035152
Galnt6	258.9247	0.359335	0.090441	3.973122	7.09E-05	0.035525
Chl1	5556.135	-0.23123	0.05833	−3.96424	7.36E-05	0.035684
Ugt8a	1119.004	0.310094	0.078628	3.943831	8.02E-05	0.036707
Enpp6	211.0008	0.386561	0.098049	3.942533	8.06E-05	0.036707
Mpp5	1289.209	−0.28844	0.073653	−3.91625	8.99E-05	0.038607
Stxbp6	915.0021	0.319802	0.081613	3.918519	8.91E-05	0.038607
Prr18	615.2873	0.347807	0.089157	3.901064	9.58E-05	0.039968
Sirt2	1240.896	0.270479	0.069584	3.887057	0.000101	0.041201

Plxnb3	797.9865	0.322193	0.083417	3.862462	0.000112	0.04438
Phldb1	2745.196	0.317234	0.082752	3.833561	0.000126	0.048655

Supplementary Table 2. Primers used for quantitative real-time PCR.

Target Gene	Primer Pairs
Foxg1	Forward: 5′-TGAGTTACAACGGGACCACG-3′
	Reverse: 5′-GTTGCCCAGCGAGTTTTGAG-3′
Gapdh	Forward: 5′-AGGTCGGTGTGAACGGATTTG -3′
	Reverse: 5′-TGTAGACCATGTAGTTGAGGTC-3′
Mal	Forward: 5′-TGTGGATCCTGATTGCCTCC-3′
	Reverse: 5′-CACTGCGGCGATGTTTTCAT-3′
Mobp	Forward: 5′-CCCCAAGGAAGAAGTGACCG-3′
	Reverse: 5′-AAAGACCCTGAGGAAGCGTG-3′
Plp1	Forward: 5′-GAGCAAAGTCAGCCGCAAAA-3′
	Reverse: 5′-GGTGGTCTTGTAGTCGCCAA-3′
Mbp	Forward: 5′-GGCAAGGTACCCTGGCTAAA-3′
	Reverse: 5′-AAATCTGCTGAGGGACAGGC-3′
Cnp	Forward: 5′-AGAGTGATCCTTGGAGCCAGA-3′
	Reverse: 5′-CGGAGGGGAATGGTGGATTT-3′
Mag	Forward: 5′-GGAGCCCAAGGGACTGTAAG-3′
	Reverse: 5′-AGGGAACTGCAATGAGGAGC-3′
Cldn11	Forward: 5′-TTGCTCTTTCCTCGGGCATT-3′
	Reverse: 5′-GGCTTCCACTGTCGTTGGTA-3′
Rpe65	Forward: 5′-TTCCCCTGCAGTGATCGTTT-3′
	Reverse: 5′-AGTTGGCTCCCCAAAGACTC-3′
Sdk1	Forward: 5′-TGTTACCCGAAGAACCACCG-3′
	Reverse: 5′-GCGGTACCTGAGGATGTAGC-3′
Gap1	Forward: 5′-GAAGTTGTGCAAGCAGTGGG-3′

	Reverse: 5′-AGGAGTGTGTGCTCTCAACG-3′
Smoc2	Forward: 5′-GGAGAGCTGGGGCAATTCTT-3′
	Reverse: 5′-CTGCAGCATGCGTTCACAAT-3′
Tenm3	Forward: 5′-AATCAGAGAGCTTGCCACCC-3′
	Reverse: 5′-TTGCTGTTGCACAAGCCG-3′
Cdkl4	Forward: 5′-TATCTGCCTTGCTTTCTTCCCC-3′
	Reverse: 5′-GTTCAGCGTTGCACCTTATTCC-3′

Supplementary Table 3. Primers used for site directed mutagenesis.

C214T_F	5′-CGCAGCAGTAGCAGCCGCCGCCGCCGCC-3′
C214T_R	5′-GCTGCTACTGCTGCGGCGGCGGCGGC-3′
C545A_F	5′-CGAGAAGCAGCCGTTCAGCTACAACGCGC-3′
C545A_R	5′-AACGGCTGCTTCTCGTACTTGCCGTTCTTC-3′
C730T_F	5′-AGGTGCCGTGCCACTACGACGACCCGGG-3′
C730T_R	5′-AGTGGCACGGCACCTTCACGAAGCAC-3′
